# Frontal Hemodynamic Responses to High Frequency Yoga Breathing in Schizophrenia: A Functional Near-Infrared Spectroscopy Study

**DOI:** 10.3389/fpsyt.2014.00029

**Published:** 2014-03-24

**Authors:** Hemant Bhargav, H. R. Nagendra, B. N. Gangadhar, Raghuram Nagarathna

**Affiliations:** ^1^Division of Yoga and Life Sciences, Swami Vivekananda Yoga Anusandhan Samsthana, Bangalore, India; ^2^National Institute of Mental Health and Neurosciences, Bangalore, India

**Keywords:** kapalabhati, schizophrenia, pre-frontal cortex, high frequency yoga breathing, fNIRS, optical topography, cerebral blood flow, near-infrared spectroscopy

## Abstract

Frontal hemodynamic responses to high frequency yoga breathing technique, Kapalabhati (KB), were compared between patients of schizophrenia (*n* = 18; 14 males, 4 females) and age, gender, and education matched healthy subjects (*n* = 18; 14 males, 4 females) using functional near-infrared spectroscopy. The diagnosis was confirmed by a psychiatrist using DSM-IV. All patients except one received atypical antipsychotics (one was on typical). They had obtained a stabilized state as evidenced by a steady unchanged medication from their psychiatrist for the past 3 months or longer. They learned KB, among other yoga procedures, in a yoga retreat. KB was practiced at the rate of 120 times/min for 1 min. Healthy subjects who were freshly learning yoga too were taught KB. Both the groups had no previous exposure to KB practice and the training was carried out over 2 weeks. A chest pressure transducer was used to monitor the frequency and intensity of the practice objectively. The frontal hemodynamic response in terms of the oxygenated hemoglobin (oxyHb), deoxygenated hemoglobin (deoxyHb), and total hemoglobin (totalHb) or blood volume concentration was tapped for 5 min before, 1 min during, and for 5 min after KB. This was obtained in a quiet room using a 16-channel functional near-infrared system (FNIR100-ACK-W, BIOPAC Systems, Inc., USA). The average of the eight channels for each side (right and left frontals) was obtained for the three sessions. The changes in the levels of oxyHb, deoxyHb, and blood volume for the three sessions were compared between the two groups using independent samples *t*-test. Within group comparison showed that the increase in bilateral oxyHb and totalHb from the baseline was highly significant in healthy controls during KB (right oxyHb, *p* = 0.00; left oxyHb, *p* = 0.00 and right totalHb, *p* = 0.01; left totalHb, *p* = 0.00), whereas schizophrenia patients did not show any significant changes in the same on both the sides. On the other hand, schizophrenia patients showed significant reduction in deoxyHb in the right pre-frontal cortex (right deoxyHb, *p* = 0.00). Comparison between the groups showed that schizophrenia patients have reduced bilateral pre-frontal activation (right oxyHb, *p* = 0.01; left oxyHb, *p* = 0.03 and right total Hb, *p* = 0.03; left total Hb, *p* = 0.04) during KB as compared to healthy controls. This hypo-frontality of schizophrenia patients in response to KB may be used clinically to support the diagnosis of schizophrenia in future.

## Introduction

*Kapālabhāti kriyā* (KB), also known as the breath of fire or the skull shining breath, involves rapid breathing consisting of active expiration with the help of abdominal muscles and passive automatic inspiration taking place during relaxation (1). Experimental data show that KB affects a broad scale of physiological processes such as respiration and cardiovascular system (2), biochemical parameters, (3) and central nervous activity (4, 5). Traditionally also, KB practice is believed to increase the blood flow to the brain (6). Immediately after the practice of KB the performance in a cancelation task (7) and auditory P300 odd-ball paradigm task (120 breaths/min for 1 min) improved (8). While performing KB, the most advocated rate of breathing is 120 breaths/min, i.e., a frequency of around 2 Hz (9).

Functional near-infrared spectroscopy (fNIRS) is a new non-invasive optical method that can measure the real time change in oxygenated hemoglobin (oxyHb) and deoxygenated hemoglobin (deoxyHb) concentrations and their sum, i.e., total hemoglobin (totalHb) or blood volume in the brain areas. Basics of the NIRS device are described elsewhere (10). An fNIRS device has excellent temporal resolution and the fNIRS results are physiologically comparable to fMRI and PET results (11). Though fMRI is a more commonly used neuro-imaging technique, we preferred fNIRS for this study because: (a) there is no interference with the yogic practice as this device does not produce any noise, (b) fNIRS is a simple to use light weight device, and (c) recordings are less expensive.

A 16-channel fNIRS device is specifically designed to assess hemodynamic responses in the bilateral pre-frontal cortices (PFCs). Studies show that dorso-lateral PFC is involved in executive tasks and attention (12), and as mentioned above, the KB practice has been used to enhance these cognitive functions; thus, its effect on pre-frontal activation was assessed in this study using fNIRS. Telles et al. (13) have recently used fNIRS device to assess the effect of KB practice on pre-frontal hemodynamic responses in 12 long term practitioners (8–36 months experience) of KB.

Studies have observed that PFC plays an important role in the pathogenesis of schizophrenia. Several research groups have found task-dependent abnormalities in frontal hemodynamics in schizophrenia (14, 15). Schizophrenia patients are being shown to have functional deficits of PFC and impaired planning ability, e.g., a study observed that during a cognitive task (tower of London) schizophrenics show poorer performance and lesser pre-frontal activation as compared to normal healthy volunteers on NIRS (16).

Reduced activation of frontal lobe during cognitive tasks (functional hypo-frontality) has been one of the most consistent findings in neuro-imaging studies of schizophrenia (17). But since this functional hypo-frontality can be influenced by various clinical factors such as psychological conditions (18, 19) and antipsychotic treatment (20, 21), its clinical significance in the diagnosis of schizophrenia has not been well established. In addition, since most of the previous studies did not have control over the task performance, a question was raised that whether the results of these studies reflect only the reduced motivation in schizophrenia. The cognitive tasks that are used to assess activation of PFCs in neuro-imaging studies have several other limitations, such as they are time consuming, costly, and require trained staff and equipment’s, which may interfere with the functioning of the neuro-imaging device. Many neuro-psychological tasks are also affected by regional and cultural factors and therefore they cannot be used in patients who belong to different culture or who are not familiar with the language in which the cognitive task is developed.

KB practice has been shown to affect pre-frontal hemodynamic responses in healthy subjects using fNIRS (13). KB is a simple physical breathing technique that does not require any equipment or skilled staff, can be taught easily, can be administered in less than 1 min, and is cost effective. Since this practice does not involve any word or symbol, it can be used on any individual who can learn the breathing practice irrespective of his geographic, educational, ethnic, or cultural background.

Thus, this study was planned with two major objectives: (1) to assess the pre-frontal hemodynamic responses to KB practice performed at 2 Hz frequency in healthy subjects and schizophrenia patients and (2) to check whether schizophrenia patients differ in their pre-frontal hemodynamic responses to KB from healthy age, gender, and education matched controls.

## Materials and Methods

### Participants

We enrolled 18 (14 males and 4 females) schizophrenia patients and 18 (14 males and 4 females) healthy subjects, who had no general medical disease, substance abuse, head injury, or diseases such as hypertension, epilepsy and ischemic heart disease where KB is contraindicated. The age of patients and controls was 28.9 ± 3.5 [mean ± standard deviation (SD)] and 26.35 ± 3.53 years, respectively, and the duration of education of patients and controls was 14.37 ± 3.8 and 15.5 ± 3.34 years, respectively (Table 1). There were no significant differences between the two groups in age, gender, and duration of education. All the subjects were right handed. Patients were recruited from inpatients and outpatients of Holistic Health Home – *Arogyadhama*, Bangalore, India and were diagnosed as having schizophrenia (14 paranoid, 2 disorganized, and 2 undifferentiated) according to DSM-IV criteria through clinical interviews by two independent psychiatrists. All patients were on antipsychotic medications by consultant psychiatrist. The duration of illness was 8.37 ± 5.6 years. All patients were medicated with antipsychotics at the examination (17 atypical and 1 typical antipsychotics) with mean chlorpromazine equivalent dosage of 289 ± 133 mg/day (22). Six out of 18 patients were medicated with anticholinergics drugs (Table 1). Both schizophrenia patients and healthy controls had no previous exposure to KB practice before (for more than a month in the last 1 year), all of them were trained by a certified yoga trainer to perform KB till they could perform it correctly (i.e., 120 ± 10 breaths/min for 1 min) and comfortably. The training was given for a period of 10–15 days before the assessments were taken. A chest pressure transducer was used to monitor the frequency and intensity of the practice. Recordings were taken only when the subject performed the practice correctly and comfortably. All subjects gave written informed consent before participating in the present study. The study was approved by the ethics committee of SVYASA University, Bangalore, India.

**Table 1:** **Demographic and clinical characteristics of subjects**.

S. no.		Healthy individuals	Schizophrenia patients	*p* Value
1	*n*	18	18	
2	Sex	14 Males, 4 females	14 Males, 4 females	
3	Age^a^ (years)	26.35 ± 3.53 (mean ± SD)	28.9 ± 3.5	0.13
4	Handedness	All right handed	All right handed	
4	Education^a^ (years)	15.5 ± 3.34	14.37 ± 3.8	0.386
5	Mean age of onset of illness (years)	–	21.68 ± 2.36	
5	Duration of illness (years)	–	8.37 ± 5.6	
6	Medications	Nil	13 Patients on resperidone alone (2 on depot preparations); 2 on resperidone plus quetiapine; 1 on aripirazole; 1 on clozapine and 1 on flupenthixol; six patients were taking anticholinergics (Tab trihexyphenidyl 2 mg BD)	

^a^Independent samples *t*-test; *p < 0.05.

### fNIRS device

The system (FNIR100-ACK-W, BIOPAC Systems, Inc., USA) is a continuous wave device, which measures changes in attenuation at two wavelengths (730 and 850 ± 15 nm), sampling at 25 kHz, and hence allows for the differentiation of two dynamic absorbers (oxyHb and deoxyHb). Equipped with 4 light emitting and 10 detector probes, 16 channels can be measured quasi simultaneously. Concentration changes in oxyHb and deoxyHb were calculated based on a modified Beer–Lambert approach (23). With an optode probe set consisting of 10 photo-detectors and 4 light emitters, 16 channels were measured. The optodes were affixed to a probe set with an inter-optode distance of 2.5 cm covering an area of ~6 cm × 18 cm. The probe set was fastened to the participant’s head bye elastic straps. For horizontal fixation, the lower edge of the probe set was fixed 1 cm above the nasion.

### Procedure

The participants reported for the assessment at morning 7:00 a.m. on different days. All recordings were taken in empty stomach, in a dark room. The subjects wore a flexible headband over pre-frontal region that contains an array of four photodiodes and 10 sensors and covered with a black cloth. The raw data were acquired from the probe, which is pre-filtered and processed in the data processing unit. The data were then sent to a laptop computer (with COBI software installed) to be digitized and read by the computer. KB was practiced by the participants at a frequency of 120 ± 10 strokes/min for 1 min, continuous recordings were taken before (5 min), during (1 min), and after (5 min) the practice.

### Statistics

Sample size was calculated using G power (24).The waveforms changes of oxyHb and deoxyHb in bilateral PFCs were acquired from all the subjects in all 16 channels and the data were averaged according to the task condition (pre, during, and post). Since spatial resolution of fNIRS device is coarse, we took the average of the oxyHb, deoxyHb, and totalHb levels on both right (channels 1–8) and left sides (channels 9–16) of the brain (13, 25). Thereby, we got one mean value of each condition (pre, during, and post) for each side of the brain (right and left) for each participant. The data were analyzed by the statistician using Statistical Package for Social Sciences version 16.0. Kolmogorov–Smirnov’s test was used to check normality of the data. As the data were found to be normally distributed, paired samples *t*-test was used to measure the changes in oxyHb, deoxyHb, and totalHb levels, respectively, during and post KB practice from the baseline (pre) levels in both the groups (schizophrenia patients and healthy controls). Independent samples *t*-test was used to compare the values between the groups. Alpha (*p* value) < 0.05 was considered to be statistically significant.

## Results

### oxyHb changes

**Figure 1:**
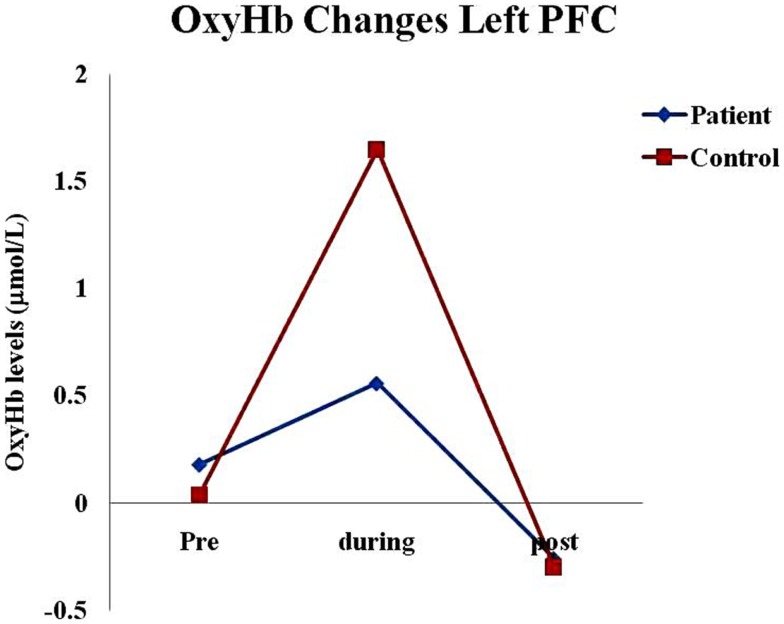
**Oxygenated hemoglobin changes in healthy subjects and schizophrenia patients in left pre-frontal cortex before (pre), during, and after (post) KB practices**. Abbreviations: oxyHb, oxygenated hemoglobin; deoxyHb, deoxygenated hemoglobin; totalHb, total hemoglobin; PFC, pre-frontal cortex.

**Figure 2:**
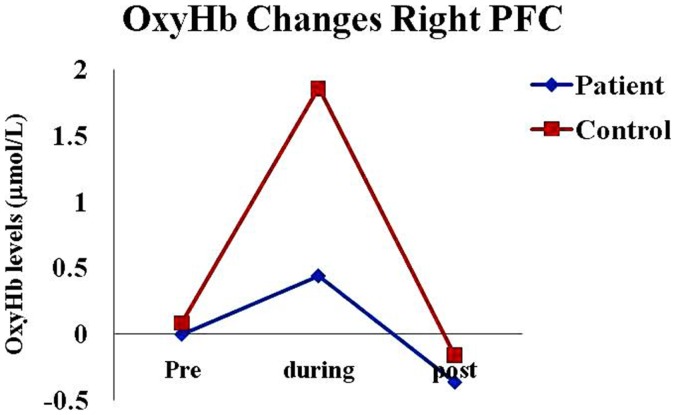
**Oxygenated hemoglobin changes in healthy subjects and schizophrenia patients in right pre-frontal cortex before (pre), during, and after (post) KB practice**. Abbreviations: oxyHb, oxygenated hemoglobin; deoxyHb, deoxygenated hemoglobin; totalHb, total hemoglobin; PFC, pre-frontal cortex.

**Figure 3:**
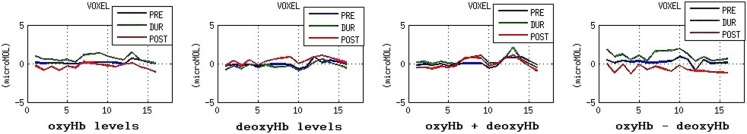
**Frontal hemodynamic responses in schizophrenia patients before (pre), during, and after (post) KB through all the 16 voxels**. Abbreviations: oxyHb, oxygenated hemoglobin; deoxyHb, deoxygenated hemoglobin; totalHb, total hemoglobin; KB, kapalabhati.

**Figure 4:**
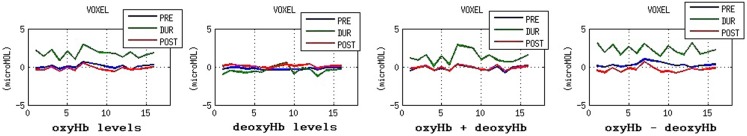
**Frontal hemodynamic responses in schizophrenia patients before (pre), during, and after (post) KB through all the 16 voxels**. Abbreviations: oxyHb, oxygenated hemoglobin; deoxyHb, deoxygenated hemoglobin; totalHb, total hemoglobin; KB, kapalabhati.

We observed a highly significant increase in bilateral oxyHb (in micromoles per liter) from the baseline during the practice of KB in normal healthy individuals; right side (*p* = 0.00) and left side (*p* = 0.00). Whereas, no significant change was found in schizophrenia patients on both the sides; right side (*p* = 0.92) and left side (*p* = 0.62) (Table 2; Figures 1–4).

**Table 2:** **Means and standard deviations of frontal hemodynamic responses before and during KB practice in patients and controls**.

Variable	Group	Side	Pre (mean ± SD)	During (mean ± SD)	Effect size	*p*^a^ Value
oxyHb (μmol/L)	Patient	Left	0.72 ± 6.17	1.48 ± 8.62	0.14	0.62
	Control		0.11 ± 4.93	9.87 ± 11.04	0.88	0.00**
	Patient	Right	−0.82 ± 3.32	−0.72 ± 5.20	0.02	0.92
	Control		0.21 ± 4.63	12.48 ± 17.29	0.78	0.00**
deoxyHb (μmol/L)	Patient	Left	1.10 ± 8.39	−0.69 ± 11.32	0.16	0.58
	Control		−1.36 ± 3.01	−2.76 ± 7.79	0.21	0.39
	Patient	Right	−0.61 ± 3.04	−3.29 ± 4.40	1.22	0.00**
	Control		−1.67 ± 3.74	−4.32 ± 8.12	0.45	0.09
totalHb (μmol/L)	Patient	Left	1.83 ± 13.19	−0.38 ± 13.40	0.08	0.77
	Control		−1.24 ± 5.01	8.10 ± 11.07	0.92	0.00**
	Patient	Right	−1.40 ± 4.06	−4.02 ± 5.15	0.60	0.06
	Control		−1.46 ± 5.21	8.16 ± 17.75	0.66	0.01*

*oxyHb, oxygenated hemoglobin; deoxyHb, deoxygenated hemoglobin; totalHb, total hemoglobin*.

^a^Paired samples *t*-test; *p < 0.05; **p < 0.01.

Between group comparisons showed that oxyHb levels were significantly higher in healthy controls as compared to schizophrenia patients during the practice of KB on both the sides; right side (*p* = 0.01) and left side (*p* = 0.03) (Table 3).

**Table 3:** **Means and standard deviations of frontal hemodynamic responses in patients and controls during KB practice**.

Variable	Side	Patient (mean ± SD)	Control (mean ± SD)	Effect size	*p*^a^ Value
oxyHb (μmol/L)	Left	1.48 ± 8.62	9.87 ± 11.04	0.84	0.03*
	Right	−0.72 ± 5.20	12.48 ± 17.29	1.03	0.01*
deoxyHb (μmol/L)	Left	−0.69 ± 11.32	−2.76 ± 7.79	0.21	0.57
	Right	−3.29 ± 4.40	−4.32 ± 8.12	0.15	0.69
totalHb (μmol/L)	Left	−0.38 ± 13.40	8.10 ± 11.07	0.69	0.04*
	Right	−4.02 ± 5.15	8.16 ± 17.75	0.93	0.03*

*oxyHb, oxygenated hemoglobin; deoxyHb, deoxygenated hemoglobin; totalHb, total hemoglobin*.

^a^Independent samples *t*-test; *p < 0.05.

### deoxyHb changes

Schizophrenia patients showed significant reduction in deoxyHb (in micromoles per liter) during the practice of KB in right hemisphere (*p* = 0.00), whereas healthy comparison subjects showed no significant change (*p* = 0.09). No change was found in deoxyHb levels on the left side of the brain in both healthy volunteers and schizophrenia patients (Table 2; Figures 3 and 4). Between groups comparison for deoxyHb changes did not show any significant difference between the two groups (control and patient) during the practice of KB (Table 3).

### totalHb changes

Significant increase was seen in bilateral blood volume (totalHb in micromoles per liter) from the baseline during the practice of KB in healthy controls; right side (*p* = 0.01) and left side (*p* = 0.00). On the contrary, totalHb levels reduced in schizophrenia patients but did not reach the level of significance; right side (*p* = 0.06) and left side (*p* = 0.77) (Table 2; Figures 3 and 4).

Between group comparisons showed that totalHb levels were significantly higher in healthy controls as compared to schizophrenia patients during the practice of KB on both the sides; right side (*p* = 0.03) and left side (*p* = 0.04) (Table 3).

## Discussion

We found that there was a highly significant increase in oxyHb and totalHb in healthy subjects during the practice of KB, which was not observed in schizophrenia patients. Schizophrenia patients showed reduction in deoxyHb in the right PFC within the group (*p* = 0.01; Table 1) but it was not significant between the groups. Frontal hemodynamic responses of schizophrenia patients to the practice of KB were different as compared to healthy age, gender, and education matched controls. Since, oxyHb is considered as the most sensitive indicator of changes in regional cerebral blood flow in NIRS measurements (26, 27), we observed that schizophrenia patients had significantly lesser PFC activation or blood flow as compared to healthy controls during the practice of KB in terms of oxyHb changes. This suggests that the effect of KB on pre-frontal hemodynamics is similar to that of a cognitive task, which can activate this region of the brain.

Some significant immediate effects of KB practice on frontal hemodynamics in long term yoga practitioners have already been demonstrated recently (13). There was a reduction in bilateral pre-frontal oxyHb and increase in deoxyHb during KB. These findings appear contradictory to what we have found. But our study differs from this study in terms of the experience of the subjects in performing KB as well as the intensity and duration of the KB practice that was administered. Study by Telles et al. (13) involved long term practitioners of KB who had 8–36 months experience of practicing KB whereas in our study we involved KB naïve subjects. Secondly, in the study by Telles et al., KB was practiced at the rate of 60 breaths/min for a total duration of 18 min in three epochs of 5 min separated by a gap of 3 min. And the average of the three epochs was taken to assess the hemodynamic responses during KB. Whereas, we administered KB for the duration of 1 min at double the frequency (120 breaths/min) and took the average of 1 min. Thus, there may be two possible explanations for the contradictory findings in the two studies: (1) subjects who were experts and were practicing KB for a longer duration may have got desensitized to this practice (similar to the practice effect seen with repeated administration of neuro-psychological tests) and thus were more relaxed during the practice thereby showing PFC deactivation instead of activation, whereas those who are performing this breathing for the first time may still remain sensitive; (2) the intensity of practice given by Telles et al. (13) was not sufficient to produce PFC activation. Pre-frontal hemodynamic responses may have a threshold below which PFC activation may not take place and this threshold may be somewhere between 60 and 120 breaths/min. In future studies, it would be interesting to assess hemodynamic responses in KB naïve subjects with gradually increasing dosage (rate of breathing) of KB and try to generate a dose–response curve along with the threshold point in oxyHb and total Hb levels.

Detailed mechanism as to how the practice of KB affects frontal hemodynamics and why schizophrenia patients respond differently from healthy subjects are yet to be understood, but as it is known that PFCs are involved in the pathology of schizophrenia (14), we may hypothesize that because of the abnormality in PFCs, schizophrenia patients have responded differently. All schizophrenia patients were taking antipsychotic medications, which make it difficult to disentangle drug effects from disease effects. A review suggested that treatment with antipsychotic medication seemed to normalize brain function and to make the brain function of schizophrenia patients more similar to that of healthy individuals (21). Therefore, the results of our study may be more because of this disease rather than the medication, although we cannot eliminate completely the medication effects. Future studies with drug naïve patients are required to discard the medication effects and confirm the findings of this study. In future studies, it would be interesting to assess and compare the hemodynamic responses to KB in patients suffering from other neuro-psychiatric disorders where PFCs are reported to be involved.

In the present study, we believe that the performance of KB practice by schizophrenia patients was comparable to healthy controls because, first, the practice was monitored objectively using a chest pressure transducer for the frequency and depth, and secondly, the patients have been adequately trained by a certified trainer and the recordings were taken only when the trainer was satisfied that the schizophrenia patients performed the practice well enough to match healthy controls. Those patients who could perform the practice as per the standards were only selected in the study. Thus, assuming that schizophrenia patients performed KB as correctly as the healthy controls, we found that the increase in frontal lobe circulation during KB is not seen in schizophrenia patients, which means that the effects of KB such as increased attention and relaxation at the subjective level (7, 8) may be lesser in schizophrenia patients as compared to healthy individuals, which we did not check in the present study. We should have assessed immediate effect of KB practice on clinical parameters and cognitive performance in schizophrenia patients simultaneously, which we plan to do in our future studies. Secondly, because of the limited number of channels, the area of measurement in NIRS was restricted to the PFC. Simultaneous measurements by NIRS and other neuro-imaging methodologies might be used to clarify the association of the PFC with other brain regions (27).

Future studies should perform a diagnostic validity of KB technique in cases of schizophrenia. Future studies should also check whether KB practice performed over a longer duration can modify pre-frontal hemodynamic responses in schizophrenia patients and whether this modification correlates with the clinical outcome.

## Conclusion

This study uses fNIRS to demonstrate that schizophrenia patients differ significantly from healthy individuals in terms of their bilateral pre-frontal hemodynamic responses to *Kapālabhāti kriyā*. Healthy individuals show significantly greater activation and schizophrenia patients show relative hypo-activation of bilateral PFCs during KB. KB practice may serve as a potential diagnostic tool to assess pre-frontal hemodynamic responses. Future studies should assess diagnostic validity of KB in schizophrenia patients.

## Conflict of Interest Statement

The authors declare that the research was conducted in the absence of any commercial or financial relationships that could be construed as a potential conflict of interest.
