# Corrigendum: Frontal Hemodynamic Responses to High Frequency Yoga Breathing in Schizophrenia: A Functional Near-Infrared Spectroscopy Study

**DOI:** 10.3389/fpsyt.2014.00055

**Published:** 2014-05-20

**Authors:** Hemant Bhargav, H. R. Nagendra, B. N. Gangadhar, Raghuram Nagarathna

**Affiliations:** ^1^Division of Yoga and Life Sciences, Swami Vivekananda Yoga Anusandhan Samsthana, Bangalore, India; ^2^National Institute of Mental Health and Neurosciences, Bangalore, India

**Keywords:** fNIRS, schizophrenia, yoga, breathing

## Author’s Description of Error and Correction

The title of Figure 4 was written as “Frontal hemodynamic responses in schizophrenia patients before (pre), during, and after (post) KB through all the 16 voxels,” which is incorrect and should be replaced with “Frontal hemodynamic responses in healthy controls before (pre), during, and after (post) KB through all the 16 voxels.”

**Figure 4:**
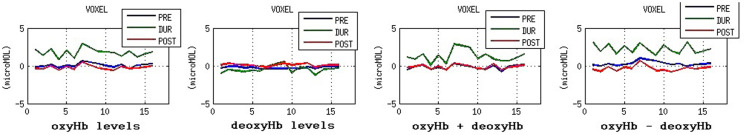
**Frontal hemodynamic responses in healthy controls before (pre), during, and after (post) KB through all the 16 voxels**.

## Conflict of Interest Statement

The authors declare that the research was conducted in the absence of any commercial or financial relationships that could be construed as a potential conflict of interest.

